# Operationalising a Recovery-Oriented Support and Information Programme Online: The EOLAS Programme

**DOI:** 10.3390/ijerph20054417

**Published:** 2023-03-01

**Authors:** Karin O’Sullivan, Carmel Downes, Mark Monahan, Jean Morrissey, Gobnait Byrne, Gerard Farrell, Patrick Gibbons, Agnes Higgins

**Affiliations:** 1 School of Nursing and Midwifery, Trinity College Dublin, D02 T283 Dublin, Ireland; 2 Trinity Centre for Practice and Healthcare Innovation, School of Nursing & Midwifery, Trinity College Dublin, D02 T283 Dublin, Ireland; 3 Kildare/West Wicklow Mental Health Service, Lakeview Unit, Naas Hospital, Craddockstown Rd., Naas, W91 AE76 Kildare, Ireland

**Keywords:** eMental health, recovery, online psychoeducation intervention, feasibility, acceptability, psychosis, service user, family/supporters

## Abstract

Evidence demonstrates that psychoeducation interventions have clinical and recovery-related benefits for people experiencing psychosis and their family members. The EOLAS programmes are one example of recovery-oriented psychoeducation programmes for psychosis. They differ from other programmes in that they are co-designed and co-facilitated (peer and clinician) group programmes. Due to the COVID-19 pandemic, EOLAS went online using a videoconferencing platform. The study examined the feasibility, acceptability and usefulness of EOLAS-Online and explored whether some of the positive recovery outcomes reported by attendees regarding the in-person programmes were replicated online. Data were collected through an online survey and semi-structured interviews. Quantitative data were analysed using descriptive statistics. Thematic analysis was used for qualitative data. Fifteen attendees (40% of attendees) completed the surveys and eight participated in interviews. A total of 80% were satisfied/very satisfied with the programme. The programme was rated highly for increased knowledge of mental health, coping strategies, and engaging with peers. The use of technology was mostly unproblematic, although some audio and video-related challenges were identified. Engaging with the online programme was experienced positively, including facilitator support to engage. The overall findings indicate that EOLAS-Online is feasible, acceptable and useful in supporting attendees’ recovery journeys.

## 1. Introduction

Primarily arising from the narratives of people who experienced mental health problems in response to their ‘dissatisfaction with the disease-diagnosis-deficit model of mental health care’ [1] (p. 43), the concept of recovery is now an underpinning principle of mental health and social care policy, including the recent [2] guidance document on the development of community mental health services. While there is much debate and critique on the meaning of recovery, with some writers commenting on how the radical intent of the original concept has been subverted and co-opted by professionals [3,4], there is little disagreement that the meaningful realisation of a recovery ethos within routine mental health care practice requires practitioners to involve service users and family members in all aspects of service design and delivery [5,6]. As a way of challenging epistemic injustice, recovery is also about embracing the collaborative sharing of information and the creation of knowledge with service users and family members [7].

Psychoeducation is an important recovery support intervention offered to people who experience psychosis (i.e., have a diagnosis of bipolar or schizophrenia spectrum disorder) and their families/supporters [8,9]. As an evidence-based intervention, psychoeducation is included in international and national treatment guidelines for psychosis [8,9]. Many systematic reviews, randomised control trials and non-control group studies provide evidence of recovery (both clinical and personal recovery)-related benefits of face-to-face psychoeducation for people experiencing psychosis and their families/supporters. Service user benefits include a reduction in the severity of distressing symptoms and relapse frequency, reduced hospital readmissions, lower self-stigma, better quality of life, improved social functioning and increased feelings of hopefulness, self-advocacy, empowerment and recovery [10,11,12,13].

Family members or close friends are also key to promoting people’s recovery journeys, as well as having their own recovery journey. The benefits of psychoeducation interventions for families/supporters include increased knowledge of mental health difficulties and supports, improved coping, better relationships between families/supporters and the person experiencing psychosis, and, reduced perceived burden and expressed emotion [14,15,16,17].

### 1.1. The EOLAS Programmes

In the Republic of Ireland, where the study was completed, psychoeducation is included in the recent model of care in relation to the care and treatment of people experiencing psychosis and their families [8]. The EOLAS (Irish for knowledge) programmes, which are the focus of this paper, are parallel group support and education programmes, one for people experiencing psychosis (and have a diagnosis of schizophrenia or bipolar disorders) and another for their families/supporters. The programmes were collaboratively developed in 2010 between a multidisciplinary community mental health team, service users, family members, voluntary agencies and academics. The EOLAS programmes were distinct from other programmes on offer at that time in that they were not provider-centred, or clinician-led like many other programmes nationally and internationally [18,19], but were co-designed and co-delivered by peers in conjunction with mental health practitioners.

The EOLAS programmes are recovery-oriented using participatory methodologies; have been co-designed and co-evaluated with service users, family members and clinicians; traditionally occur through in-person group format; and are co-facilitated by a peer and clinician. While not aligned with a particular theory of recovery, the programmes are underpinned by principles of adult education and are aimed at enabling attendees to increase their knowledge about psychosis, in support of the person’s recovery journey. The manualized programmes comprise eight weekly group modules of 90 min duration, which explore the topics of psychosis, biopsychosocial treatment options, accessing services and support, and dealing with stigma and self-advocacy. For people experiencing psychosis, the programme also explores methods of dealing with voices and distressing beliefs, maintaining recovery and preventing relapse, and outlines people’s rights and entitlements. Additional family/supporter-specific modules explore how mental health difficulties affect families/friends, coping and effective communication, the family in recovery and planning for the future. In the ethos of collaboration, potential participants, service users and families/supporters, hear about the programmes through clinical teams and have an opportunity to discuss suitability and relevance before deciding to attend. EOLAS programmes have been rolled out in 15 mental health services nationally.

EOLAS has been evaluated on an ongoing basis using participatory methodologies that have included quantitative and qualitative approaches, as well as through the use of PhotoVoice, the latter being a participatory action research methodology; a process of collaboration, empowerment, and equal partnership, where participants communicate their lived experiences through photography, in a context of shared understanding among project members that expertise is located with people with lived experience of the topic at hand [20] (p. 633). Quantitative data from pre-post design surveys demonstrated significant improvements in knowledge about mental health issues, recovery attitudes, and sense of hope and confidence following programme participation. Participants in follow-up interviews also reported a greater understanding of the recovery process, self-care, increased communication with mental health teams, increased awareness of communication patterns within the family unit, a reduced sense of isolation, and increased social networking. The co-facilitation model was regarded positively, with the presence of peer facilitators being viewed as inspirational and generating hopefulness [21,22,23].

The COVID-19 pandemic restrictions necessitated the suspension of in-person EOLAS programmes. In early 2021, funding became available to modify the manualized EOLAS programme and pilot a real-time online format using a videoconferencing platform. In the context of Ireland that was timely as there was a growing consensus that health systems needed to exploit technologies to improve health provision [24,25], with the recently published national Mental Health Policy [26] recognizing the potential for technological solutions to meet people’s needs for convenient, affordable, and readily accessible mental health information and supports.

To prepare for the change in the mode of delivery, peer and clinician facilitators received training for online facilitation, delivered by training consultants with experience of EOLAS facilitation training for the in-person programme. Training comprised three blocks: (a) EOLAS-Online Facilitators Programme; (b) Using Online Tools and Platforms; and (c) Reconfiguring EOLAS for online delivery. Once facilitator training was completed, facilitators went on to deliver the EOLAS Programmes at two pilot sites. This paper presents findings on attendees’ (service users and family members) experiences of attending the EOLAS-Online pilot programme.

### 1.2. Virtual Interventions

**Figure 1:**
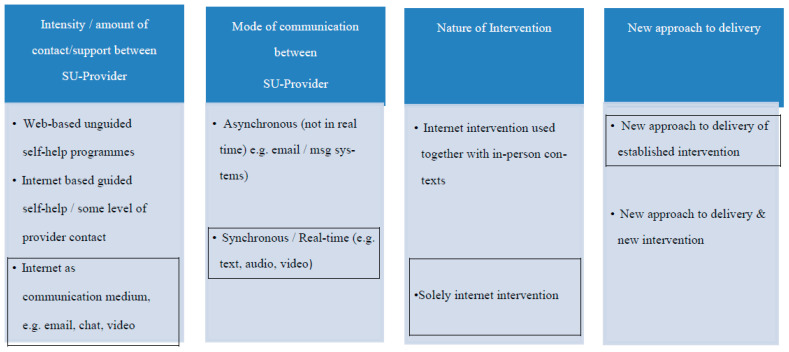
A typology of virtual health options. Source: Adapted from Berger, 2017.

Virtual interventions are highly variable. Berger’s [27] typology of four distinguishing criteria provides an overview of this variability (Figure 1). The criteria range across the intensity of contact and support between service users and providers, how communication occurs, whether the digital intervention has in-person components, and whether the innovation is through a novel mode of delivery of established interventions or is itself a new model of intervention. In the context of Berger’s [27] typology, EOLAS-Online is a programme where the internet is the communication medium of a synchronous/real-time, solely internet intervention, and is a new approach to the delivery of an established initiative (as outlined in Figure 1).

The literature on digital mental health interventions for people with serious mental health problems also suggests a varied landscape. Psychoeducation digital interventions in particular have been designed for people with a range of diagnoses, for example, people with a diagnosis of bipolar disorder [28,29,30], schizophrenia [31,32,33,34] or first-episode psychotic or mood disorder [35], while others focus on people with ‘serious mental illness’ [36]. Among the interventions identified for family/caregivers of people experiencing psychosis are programmes focused on caregivers of people with psychosis [37], first-episode psychosis (FEP) [38], long-term psychosis [31,39], and siblings of people with psychosis [40]. Interventions may also be aimed at both people with psychosis and their families/support persons [33,34].

In terms of contact between service user and provider, interventions ranged from internet-based self-guided programmes with no practitioner/provider contact [30,41] to internet self-guided programmes with clinician/provider contact [28,29,32,35,36,42,43]. Programmes used combinations of asynchronous [28,29,33,34,35,37,39,40,43], and/or synchronous modes [31,36,42] of communication.

Most were delivered via an online platform [28,29,30,31,32,33,34,35,38,40,43] with some designed specifically for delivery via smartphone [36,42]. In terms of peer engagement, while some of the interventions incorporated a peer element [31,35,37,39,41], the level of involvement of peers in the design or co-facilitation of the programmes was unclear, with many programmes being led or moderated by a clinician.

Reviews of online psychoeducation interventions have not only found them to be acceptable and usable but report that they have the potential to improve psychotic experiences/symptoms, coping skills, hospital admission rates, social connectedness, depression and medication concordance [44,45,46], with many of these positive outcomes being equivalent, and some better, to those in usual care contexts.

Systematic reviews on digital interventions for families/supporters of people with mental health problems have found them to be promising in terms of their feasibility, acceptability and satisfaction [47,48], with one review [47] reporting improvements in family members’ experiences (improved knowledge of psychosis, support for sharing of experiences, and reduced perceived burden and stress).

Reviews specifically on the use of videoconferencing with people with serious mental health problems and their families found that this forum is generally feasible and acceptable, with high rates of service user satisfaction, and mainly equivalent findings between in-person and online conditions [49,50]. However, a concern raised in both reviews was an individual’s potential for ‘delusions of reference’ (a symptom of psychosis), which might impact negatively on the use of videoconferencing. Other challenges identified included the need for repeating instructions, higher levels of fatigue than in-person interactions due to prolonged screen exposure, technical, audio and visual challenges [49] as well as poor quality bandwidth [50]. However, the studies included in these reviews are different from EOLAS-Online as none of the studies used a videoconferencing platform for real-time programme co-facilitation.

## 2. Methods

### 2.1. Aim of the Study

The aim of this study was to examine the feasibility, acceptability and usefulness of EOLAS-Online and to examine if some of the positive outcomes reported with the in-person programme were replicated online.

### 2.2. Design

A sequential design was used involving the collection of quantitative data through an anonymous online survey (Qualtrics, 2021), followed by qualitative data via semi-structured interviews (telephone/videoconferencing) (KOS).

### 2.3. Data Collection Tools

The survey and semi-structured topic guide were designed by the research team with the involvement of a person with lived experience of psychosis and a family member. The survey had five sections examining: (i) motivation and usefulness (11 questions), (ii) online experience of the programme and using the technology (20 questions), (iii) overall satisfaction of the online programme and its impact (9 questions), (iv) number of/reasons for missing sessions and suggestions for improvement (12 questions), and (v) background information and demographics (3 questions). Both closed and open-ended questions were used. The closed questions were mostly Likert scale-type questions on a scale from 1 (strongly disagree) to 5 (strongly agree). While some of the questions were drawn from previous questionnaires used to evaluate the in-person programme, the majority of the questions were orientated towards the online experience.

The semi-structured interview topic guide inquired into attendees’ personal experience of the programme content, peer involvement, the online forum, challenges encountered, and suggestions for improvement. Interviews were audio-recorded.

### 2.4. Setting

Undertaken on the Webex videoconferencing platform, the EOLAS-Online pilot programme occurred in two mental health sites that had previously run the in-person programme and volunteered to pilot the online format. Site one comprised four groups (two service users, two family members/supporters), and site two had two groups (one service user, one family member/supporter). Programmes ran between October 2021 and February 2022. Similar to the in-person programme, attendees were informed about the programme by clinicians and suitability was discussed. A criterion for participation in the pilot was that attendees had access to the necessary hardware, i.e., smartphone, tablet, or computer and internet access. Programme co-ordinators at each site posted hard copies of the relevant EOLAS Handbook to attendees with instructions for accessing the videoconferencing platform and an access link. Group facilitators provided support for using the online platform from that point onwards.

### 2.5. Recruitment to the Evaluation

Clinical facilitators acted as gatekeepers, distributing an information sheet and a link to an explanatory video to attendees at the start of the programme. These outlined the purpose of the research, the mode of data collection, and the ethical principles of the study. At the penultimate and final sessions of the programmes, all attendees were provided with a link to the survey and invited to complete it at the end of the programme.

Attendees were alerted to the option to opt-in to the qualitative component at the end of the survey by ticking the relevant box, which directed them to a Microsoft Forms opt-in page and invited them to provide their contact details. Attendees were then contacted by a researcher (KOS), participation was discussed, any questions answered, and a time and venue for undertaking the interview were arranged.

### 2.6. Data Analysis

Quantitative data were analysed using IBM SPSS Statistics for Windows (Version 27) (Armonk, NY, USA). (CD). Categorical data are summarized using frequencies (*n*) and percentages (%); continuous data are summarized using means (M) and standard deviations (SD). A mean closer to 5 on the scale for positively worded items and closer to one for negatively worded items indicated more positive evaluations.

Interviews were transcribed verbatim, entered into NVivo (Release 1.5), (QSR International Pty Ltd., Burlington, MA, USA, 2021), and analysed using a thematic approach [51]. The data were open coded initially (KOS, AH, MM, JM, GB, GF), compared for similarities and differences, and then grouped into broader categories and themes (KOS). Codes utilized were SU for service user and FM for family member.

### 2.7. Ethics

Ethical approval was received from the Faculty of Health Sciences Research Ethics Committee at Trinity College, Dublin (#210904), and from the two regional ethics committees for the mental health service pilot sites. Attendees were informed that participation in the evaluation was voluntary, and that non-participation would not adversely affect their participation in the EOLAS-Online programme or access to clinical care. Participants were asked to indicate their consent prior to participating in the survey using a tick box option and were advised that they could exit the survey at any point without submitting it. Interview participants digitally signed and returned an online consent form prior to the interview. Interviewees were advised that they could choose to not answer any of the questions and end the interview at any point.

## 3. Results

### 3.1. Participant Profile

In total 37 attendees (Service User *n* = 16, Family Member *n* = 21) participated across the six online pilot programmes. Of these attendees, fifteen (40.5%) participated in the survey, and eight (21.6%) participated in the interviews. At a role level, the survey response rate was 56.2% (9/16) for service users and 28.5% (6/21) for family members. These details were missing for one participant (Table 1).

Of the 15 participants who completed the survey, 53.3% (8/15) identified as service users, 33.3% (5/15) as a family member or friend and one person (6.6%) as both. Most survey participants (*n* = 10, 66.6%) were female and four were male, with an average age of 45.1 years (*n* = 14, Range = 26–66, SD = 12.6). Eighty percent (*n* = 12) had no prior experience of participating in an online support or education group. Of the eight attendees that took part in telephone interviews, six identified as service users and two as family members. Most interviewees (*n* = 6, 75%) were female (Table 1).

Approximately two-fifths (40%, *n* = 6) of the survey sample indicated that they did not miss any of the eight EOLAS-Online sessions. Of the nine that missed sessions, seven indicated that they missed just one while two attendees reported that they missed three. For the majority (88.9%, *n* = 8), time conflicting with other things was the main reason for not attending some sessions, with attendees citing work commitments, healthcare appointments, and holidays as some of the specific reasons. It is noteworthy that none of the attendees identified issues relating to the programmes themselves, such as technological difficulties, privacy/confidentiality concerns, irrelevant material, or dissatisfaction with interactions within the group as reasons for missing sessions (see Table 2b).

### 3.2. Impact of the Programme

The impact of EOLAS-Online was found to be positive in terms of attendee ratings of knowledge of mental health (M = 4.60, SD = 1.06, *n* = 15), coping strategies (M = 4.40, SD = 1.12, *n* = 15), and the benefits for them of meeting other people with similar experiences (M = 4.47, SD = 1.13, *n* = 15) (Table 3a). These three items were also among attendees’ highest-rated motivations for attending the programme, (i.e., *n* = 14/15, *n* = 15/15, and *n* = 15/15, respectively) (Table 2a above).

Programme impact (Table 3a) was also found to be positive in terms of self-care (M = 4.20, SD = 1.15, *n* = 15), knowing more about how to get mental health-related support (M = 4.47, SD = 1.06, *n* = 15) and sharing experiences to help others (M = 4.27, SD = 1.16, *n* = 15). The lowest scoring impact item was the statement ‘*I have increased my social network*’ (M = 3.40, SD = 1.18, *n* = 15).

Overall attendee ratings of programme usefulness (M = 4.47, SD = 0.74, *n* = 15), and satisfaction (M = 4.20, SD = 1.2, *n* = 15) were high (Table 3b), with the majority of attendees reporting that they would recommend the online programme to others (86%, *n* = 13).

Interview findings provided some further programme impact-related context. For example, this attendee reported having taken back some control over her life, an experience that emerged during participation in group exercises.

“*I made myself [goals]. For me it was… to get up early in the morning… Now obviously being off work and stuff I just fell into a rut, so it was just managing my time better in the morning. Getting up at seven and getting lunches prepared and stuff. And to get me back exercising. And since then, I have been [doing these]. Yeah, yeah, I feel like I’ve taken back a bit of control over myself*.”

(8003, SU)

Attendees also spoke positively of the recovery impact of being with peers, and for some, this was identified as being the most important part of the programme. Attendees collectively associated this with feeling less isolated, more supported and understood, gaining a better self-understanding of mental health issues, being better able to support relatives, and increased hope for the future.

“*And just in general… talk…, be able to talk to somebody, a peer, about your condition which was very helpful, yeah. Well, it allowed me as the weeks went on to be more open about my own condition and how it affected me. And yeah, by the last couple of weeks I was talking clearly and able to share my stories*.”

(8004, SU)

“*So, it was nice to be able to say that yeah people in all, like people in all walks of life have it [mental illness] and that we can still function and have a good job as well because of it*.”

(3001, SU)

Additionally, according to this family member,

“*You learn how some people are coping, what challenges they have, how they are managing them and… you know, it gives you a bit of insight into what [services] is out there*.”

(4001, FM)

However, for one person, a sense of decreased hope was associated with the realization that many other family member attendees were supporting people with severe and long-term psychosis.

“*While I was new to experiencing a family member suffering psychosis, I felt that the other participants’ family members had severe and long-term psychotic mental illness. From this I was left feeling isolated and a little hopeless at the prospect of recovery. But that did not mean I didn’t take good information from the sessions*.”

(FM, survey comments)

### 3.3. Use of Technology and Technology Support

Survey results suggested that overall, attendees found the use of technology unproblematic (Table 4a). Respondents rated highly their level of confidence in the safety of the platform (M = 4.67, SD = 1.05, *n* = 15), ease of use (M = 4.60, SD = 1.10, *n* = 15), ease of joining the sessions (M = 4.60, SD = 1.06, *n* = 15), technical capacity of their own device to facilitate full engagement in the sessions (M = 4.27, SD = 1.16, *n* = 15) and ability to share their video feed (M = 4.13, SD = 1.25, *n* = 15). Specific advantages identified in the interviews included the flexibility of being able to log on from anywhere, not having to travel, being able to attend from one’s own home, having the option of anonymity, and accessibility for attendees with child-minding responsibilities. Notably, 66.7% of attendees (*n* = 10) identified convenience due to not having to travel, as a motivation for attending EOLAS-Online (Table 2a).

For some negatively-worded statements under the theme of Use of Technology (Table 4a), slightly higher scores (i.e., indicating a more negative experience) were noted: ‘I found it difficult to hear what people were saying’ (M = 2.07, SD = 1.28, *n* = 15), and ‘I found it difficult to see people and read their non-verbal cues’ (M = 2.27, SD = 1.28, *n* = 15).

Interviews provided some insight into these scores. For one attendee who used a mobile phone, a degree of detachment from the interactions online was experienced:

“*You wouldn’t be as into it [in comparison to in-person communication]. …there was just, ‘Am I talking or am I not talking or am I being heard, or can you see me?’ There was a bit of that going on*.”

(9001, FM)

Some attendees also indicated challenges in interacting with people who chose not to turn on their cameras:

“*The challenges were mainly the people who didn’t show their face online. It was hard to interact with those people*.”

(8004, SU)

However, these negative experiences could not be generalized, as some other attendees who also used a mobile phone, did not experience non-camera use as a difficulty:

“*No. I didn’t have an issue with it [non-camera use]. I don’t think anyone did really … for the people were still talking and sharing experiences. …there was no negative impact in any way…*.”

(8002, SU)

Half of the survey attendees ‘strongly agreed’ with the statements ‘I found the technical support helpful’ (50%), and ‘any technical problem I had was resolved quickly’ (45.5%), while a minority (10% and 18.2%, respectively) ‘strongly disagreed’ with the latter statements. Many attendees were undecided about these statements (40% and 36.4%, respectively), which could reflect that technical issues had not arisen for them or that they were able to troubleshoot any difficulties without the need for technical support. Interview data also indicated that some technical issues arose, but that facilitator support was available to attendees for resolving these problems.

### 3.4. Experiences of Attending EOLAS-Online

Attendees generally indicated positive experiences of engaging with EOLAS-Online (Table 4b) and reported being comfortable speaking online (M = 4.47, SD = 1.13, *n* = 15), the content met their needs (M = 4.27, SD = 1.39, *n* = 15), were comfortable sharing their video feed (M = 4.00, SD = 1.51, *n* = 15), had access to a private space/room for participating in the sessions (M = 4.40, SD = 1.30, *n* = 15), and had a positive experience of facilitator support to engage (M = 4.40, SD = 1.40, *n* = 15). The qualitative findings confirmed this conclusion, with only one negative experience of facilitation reported, relating to instances where repetitive contributions by group members were not perceived to be managed well.

Attendees’ suggestions for improvements included more time allocation to attendees talking amongst themselves, the use of some in-person sessions, consideration to the time-of-day of the programme (time-of-day being the only reason identified for missing sessions—Table 2b).

Notably, no attendees reported missing sessions because of concerns about the privacy and confidentiality of the online forum (Table 2b), with attendees indicating a low level of mistrust in relation to online platforms (M = 1.20, SD = 0.41, *n* = 15) (Table 4b). Interview findings indicated that addressing the issue of safety and privacy is important especially early in the programme. Early discussion of ground rules can alert attendees to safety and privacy issues and implications they may not have previously considered:

“*Just when they said about recording, I hadn’t thought of it before. Only when [the co-facilitators] said it I kind of thought, ‘Oh yeah, people could record that now and put it somewhere else’, you know*.”

(3002, SU)

In addition, one attendee raised concerns regarding potentially knowing participants on the programme who might disclose to others about their diagnosis:

“*I was just afraid I’d know someone on the course that would tell people [e.g., work colleagues] like that I’m bipolar. So, you know yeah, yeah it’s always kind of in the back of your mind*.”

(3001, SU)

## 4. Discussion

The study findings indicate that EOLAS-Online is feasible and acceptable for people who experience psychosis and their families/supporters. The recovery benefits of EOLAS-Online were evident in terms of the reported improved knowledge of mental health, coping strategies, meeting people with similar experiences, self-care, knowing where to get support, and sharing with others. While the findings are not directly comparable to evaluations conducted on the in-person programmes, the high rates of satisfaction and usefulness in terms of knowledge acquisition do correspond closely to positive impacts identified for the in-person programme, where statistically significant higher levels of knowledge in relation to mental health issues and coping strategies were found in the pre-post evaluations [21,22]. Together with the qualitative findings these impacts aligned with the five recovery processes (identified by Leamy et al., [52]) of connectedness, hope and optimism about the future, identity, meaning in life, and empowerment, although the endorsement of attendees in relation to having increased their social network was relatively weak. This latter finding can be contrasted with qualitative findings from the in-person programmes, which found that, particularly for families, peer contact continued after the programme ended and was an important benefit of attending the EOLAS in-person programmes [23]. The value of sustained contact between peers is recognized in other research that compares the continuity of relations between online and in-person environments [53]. Thus, exploring ways to facilitate peers to remain in contact post-programme completion may be of value in future iterations of EOLAS-Online.

Overall, the use of the technology was not found to be challenging for attendees, although some problems were encountered with specific devices (particularly smartphones) in relation to seeing, hearing and conversing with group members and the use/non-use of cameras. However, different perspectives on these issues were evident, suggesting, in keeping with Santesteban-Echarri et al., [49] that individual variables and attitudes may be more relevant than technical characteristics of the equipment used for people experiencing communication challenges via videoconferencing. The advantages of the online context identified included flexibility, accessibility, and inclusiveness for attendees with child-rearing responsibilities. This finding is important given the studies that highlight that despite the successful integration of psychoeducation into policy recommendations and clinical guidelines, equitable access for many service users and family members is not always achieved [54,55].

Internet connectivity problems were not identified as an issue among EOLAS-Online attendees; however, a criterion for participation was that attendees had appropriate devices and access to the internet. In terms of equity of access, although Ireland has a National Broadband Plan to deliver high-speed broadband to all premises in Ireland [56], progress to date has been slow [57]. Uneven implementation means that broadband speeds can be highly variable depending on where one lives [58]. Internet connectivity, including bandwidth, would therefore require consideration, should the EOLAS-Online programmes be extended beyond the pilot. Indeed, Watson et al., [59] suggest that poor internet speed is among the reasons offered by participants in their study for declining to take up remote/videoconferencing therapy. In relation to the expansion of the online programme, the question of digital literacy and inequality of access would require consideration to ensure that those who do not have access to appropriate devices or the internet are not excluded from participating. It would also be important that the EOLAS in-person programmes continue, to avoid the programme becoming a contributor to the digital divide.

It is reassuring that none of the service user participants in this research indicated that they had experienced an exacerbation of psychotic symptoms during their participation in EOLAS-Online. In addition, no attendees who registered to participate in EOLAS-Online, but did not take up their place, identified this concern as the reason for not attending. While this is in line with findings in the literature [49,50], more recent research has found that voices and unusual experiences or beliefs contributed to people with psychosis declining to engage in video conferencing remote therapy [59], highlighting the need for continued awareness of this possibility.

Safety and privacy are key considerations for service users with severe mental health problems [60]. In the main, safety and privacy concerns were not an issue in this study, but the findings did highlight the pre-programme information and inclusion in the first module as particularly important stages for consideration of these issues.

### Limitations

While the findings provide important insights for the future development of the EOLAS-Online programme and add to discussions on digital technologies within the mental health space, it is important that they are read in the context of the following limitations. This evaluation is based on the view of 40% of those who participated in the programme; therefore, the findings may only reflect those who were more positive about the programme, had high levels of trust in technology and had high digital literacy. Social desirability bias may also be an issue for those who did participate, as they may not have wanted to be critical of the programme in case the online format was discontinued and not made available to others. As stated, one of the inclusion criterion for participating in the pilot was that participants had to have internet access and an appropriate device, which may have ruled out people with less digital literacy and living in areas with poor access to the internet or variable broadband speeds.

## 5. Conclusions

There have been moves to include the use of the online environment for psychoeducation interventions for people who experience psychosis and their family members. This study adds to this literature on online psychoeducation interventions in reporting on EOLAS-Online; a real-time, co-facilitated set of parallel group programmes via videoconferencing, for people experiencing psychosis and their families/supporters. Notwithstanding the limitations, this study provides some evidence, based on attendees’ experiences, for the feasibility, acceptability and utility of EOLAS-Online in supporting people’s recovery journeys, which is a pilot online version of an established in-person programme.

Future research should examine provider perspectives on online programme implementation to explore their views and to identify if any specific concerns arise for them. Further research of this population and their supporters with a larger sample is warranted that examines online accessibility to ensure that EOLAS-Online, and other similar programmes, do not contribute to the digital divide.

## Figures and Tables

**Table 1:** Sociodemographic characteristics of participants.

	Survey Participants (*n* = 15)	Interview Participants (*n* = 8)
Role	*n*, (%)	*n*, (%)
Service user	8 (53.3%)	6 (75%)
Family/Supporter	5 (33.3%)	2 (25%)
Both	1 (6.6%)	-
Missing	1 (6.6%)	-
Gender		
Female	10 (66.6%)	6 (75%)
Male	4 (26.6%)	2 (25%)
Missing	1 (6.7%)	-

**Table 2:** Motivations and reasons for missing a session.

(a) Motivations for Attending EOLAS-OL *	***n***	**%**
Learn more about coping strategies	15	100%
Meet other people with similar experiences	15	100%
Increase my knowledge of mental health	14	93.3%
Share experiences to help others	12	80%
Learn more about self-care	10	66.7%
Get support for my mental health	10	66.7%
The online programme was more convenient as did not have to travel so saved time	10	66.7%
Increase my social network	6	40%
(b) Reasons for Missing Sessions *	**No**	**Yes**
Time conflicted with other things	1	8
Session was not relevant to my needs	9	0
Didn’t feel my voice was being heard	9	0
Found other people’s stories too upsetting	9	0
Found the technology too difficult to navigate	9	0
Internet connection was poor	9	0
I had concerns about the privacy and confidentiality of the online forum	9	0
I didn’t have access to a private computer at the time the session was on	9	0

* tick any that apply.

**Table 3:** Impact, Usefulness, Satisfaction.

**(a) Impact (*n* = 15)**		
Please rate your level of agreement with the following statements about the impact of EOLAS? (*n* = 15)	Mean	Std. Deviation
EOLAS increased my knowledge of mental health	4.60	1.06
I know more about where to get support for my mental health	4.47	1.06
It was beneficial to share experiences to help others	4.27	1.16
I learnt more about coping strategies	4.40	1.12
It was good to meet other people with similar experiences	4.47	1.13
I learnt more about self-care	4.20	1.15
I have increased my social network	3.40	1.18
**(b) Overall Measures**		
Usefulness (*n* = 15)	4.47	0.74
Satisfaction (*n* = 15)	4.20	1.2

**Table 4:** Attendees’ experiences of (**a**) using technology, (**b**) doing EOLAS-OL.

(a) Please Rate Your Level of Agreement with the Following Statements about Using Technology to Do EOLAS Online? (*n* = 15)	Mean	Std. Deviation	(b) Please Rate Your Level of Agreement with the Following Statements about Your Experience of Doing EOLAS Online? (*n* = 15)	Mean	Std. Deviation
I had confidence in the safety of the platform being used	4.67	1.05	I was comfortable speaking online	4.47	1.13
I found the technology easy to use	4.60	1.10	The handbook complemented the session content	4.40	1.12
I found it easy to join the session	4.60	1.06	I always had access to a private space/room	4.40	1.30
I have a good/stable internet connection where I live	4.40	1.12	The facilitator provided sufficient encouragement/opportunity for me to engage	4.40	1.40
My computer enabled me to fully engage in the session	4.27	1.16	The content of the material met my needs	4.27	1.39
I was able to share my video	4.13	1.25	I was comfortable sharing my video feed	4.00	1.51
I found it difficult to hear what people were saying	2.07	1.28	I found the online platform was not conducive to sharing personal experiences	1.87	1.30
I found it difficult to see people and read their nonverbal cues	2.27	1.28	I don’t trust online platforms	1.20	0.41

## Data Availability

Data not available for privacy and ethical reasons.
